# Optimism Bias Among Gun Owners: Associations With Firearm Injury Prevention Practices and Policy Support

**DOI:** 10.1177/10901981241267212

**Published:** 2024-07-30

**Authors:** Amanda J. Aubel, Garen J. Wintemute, Aaron B. Shev, Nicole Kravitz-Wirtz

**Affiliations:** 1 Violence Prevention Research Program, Department of Emergency Medicine, School of Medicine, University of California, Davis, Sacramento, CA, USA

**Keywords:** injury prevention, firearm, policy, public health, optimism bias, risk perceptions

## Abstract

Optimism bias is common across health risk assessments, including firearm injury risk, and can have behavioral consequences. Using data from the 2018 California Safety and Wellbeing Survey, we examine whether optimism bias influences firearm injury prevention practices and policy support by comparing the characteristics, behaviors, and opinions of gun owners who believed having a gun at home is comparatively safer for themselves than for similar others (*Optimism Bias* group) with (1) those who unequivocally believe guns increase safety for themselves and others (*Always Safer* group), and (2) those who said they “don’t know” or “it depends” in both the self and other scenarios (*Uncertain* group). Weighted multinomial logistic regression results indicated that gun owners in the *Optimism Bias* group were more often female, members of minoritized racial or ethnic groups, and new gun owners than the *Always Safer* and *Uncertain* groups; they also demonstrated greater support for 4 of 5 firearm injury prevention policies/interventions. Despite similar prevalence of owning a gun for protection, gun owners in the *Optimism Bias* group less often carried a loaded firearm or stored a gun in an unsecure way compared with the *Always Safer* group. Findings suggest that gun owners characterized by optimism bias, who acknowledged some risk associated with firearms, even if only or more so for others than for themselves, may represent a “movable middle” that is more receptive to firearm injury prevention efforts. Public health messages emphasizing other-oriented (vs. personal) risk and collective responsibility may be perceived as less threatening to the symbolic significance of guns to individual identity, thus enhancing effectiveness.

## Introduction

A substantial body of scientific evidence finds that having a firearm at home increases the risk of firearm death and injury for everyone living in the home (Anglemyer et al., 2014; Hemenway, 2011; Studdert et al., 2020). Despite this well-documented threat to health and safety, a large and growing number of people own firearms because they believe that having a gun at home will protect them from harm. In the United States, nearly two in three gun owners (63%) cite self-protection as a primary reason for ownership; among those who own handguns, this percentage is even higher: 76% in 2015 (Azrael et al., 2017). For comparison, just two decades earlier, less than half of handgun owners (48%) reported self-protection as their primary reason for ownership (Cook & Ludwig, 1997).

Risk perceptions—or a person’s beliefs about their own and other people’s vulnerability to (or risk of) harm—play a central role in theorizing about health-related decision-making and behavior change. In the case of firearm ownership, it is reasonable to expect that a person’s decisions about whether to own a firearm and whether to engage in protective behaviors that could mitigate firearm injury risk (e.g., keeping all firearms unloaded and locked up when not in use) are shaped by psychological heuristics about the likelihood of resultant negative and positive occurrences, such as firearm injury and death or defense of self, family, and property (Hoskins et al., 2020). However, research suggests that misperceptions about the risks and benefits of firearm ownership are common.

In California, for example, individuals living in homes with firearms, including both owners and non-owners who live with owners, are 50% to 60% less likely to worry about personal firearm injury than people living in homes without firearms (Schleimer et al., 2021). Nationally, adults in firearm-owning households tend to believe that unintentional (“accidental”) firearm injuries occur more frequently than intentional self-inflicted or assaultive firearm injuries, even for subgroups of people characterized by elevated levels of risk for harming themselves or others on purpose (Rowhani-Rahbar et al., 2022). National survey findings further indicate that fewer than 10% of gun owners (and a similar percentage of non-owners who live with owners), and only 20% of people in homes without guns, associate household firearms with increased suicide risk, despite evidence to the contrary (Conner et al., 2018). This same survey also found that nearly 60% of gun owners report that guns make homes safer and, as a group, these owners are more likely to store household firearms loaded and unlocked, a well-established risk factor for firearm injury (Grossman et al., 2005; Mauri et al., 2019).

While past research has drawn attention to firearm-related risk perceptions in absolute terms (i.e., the perceived likelihood of firearm injury occurrence), assessments using comparative measures (i.e., the perceived likelihood of a firearm injury occurring to oneself compared to someone else) have been more limited. This gap persists despite a robust literature indicating that people tend to conceptualize risk differently when they are asked to rate risks with regard to themselves versus with regard to other people in general (Hoorens, 1994). Specifically, people tend to be unrealistically optimistic about the risks and benefits they will personally experience in relation to their own actions when compared with the risks and benefits other people will experience when undertaking the same actions.

This tendency, termed “optimism bias” (or “unrealistic optimism”), has been observed across numerous health risk assessment scenarios, including when diverse subgroups of people estimate that they are at lower risk of being victims of a heart attack, a motor vehicle crash, smoking-induced cancer, a sexually transmitted infection, and a hazardous event at work compared with most others (Caponecchia, 2010; Hoorens, 1994; Rise et al., 2002; Weinstein & Klein, 1995; White et al., 2004). Practically speaking, however, except in very skewed population distributions, it is impossible for most members of a collective group to be better off—for example, less likely to experience a firearm injury or more likely to use a firearm to deter a crime—than all other members of that same population. Furthermore, optimism bias has been shown in past research to impact the extent to which individuals are willing or likely to engage in precautionary health practices, such as masking to prevent COVID-19 infection and screening for breast cancer, and to support health promotion policies more generally (Fragkaki et al., 2021; Katapodi et al., 2009).

In the context of increasing firearm ownership for protection-related reasons, this general population survey study of adult residents of California describes the prevalence of and factors associated with comparative risk perceptions among gun owners in the state. Specifically, we examine whether gun owners with optimism bias differ from gun owners with other patterns of risk perceptions in their sociodemographic characteristics, firearm ownership behaviors, and support for selected firearm injury prevention policies and interventions. Findings from this analysis may helpfully inform the effectiveness of firearm injury prevention communications and, ultimately, contribute to reductions in firearm injury and death.

## Method

### Data

Data came from the 2018 California Safety and Wellbeing Survey (CSaWS 2018), a statewide, repeated cross-sectional, web-based survey designed by our research group and administered in English and Spanish in Fall 2018 by the survey research firm Ipsos. CSaWS 2018 collected detailed information on firearm ownership and use, exposure to violence and its consequences, and public opinion on firearm violence prevention policy. The sample comprised members from the Ipsos Knowledge Panel (KP), a national probability-based research panel. All KP members residing in California households who were aged 18 years or older were eligible to participate. Of the 5,232 eligible members invited to participate, 2,558 completed the survey (response rate, 49%), of whom 429 were gun owners (14% of the adult population of California). Additional details about CSaWS 2018 methodology and sample characteristics are in the project’s initial report (Kravitz-Wirtz et al., 2020). CSaWS 2018 was approved by the Institutional Review Board at the University of California, Davis.

### Measures

#### Optimism Bias and Other Risk Perception Patterns

To assess firearm-related risk perceptions, we asked respondents two questions: (1) “Does having a gun at your home make it a safer place to be, or a more dangerous place to be?” and (2) “If everyone in your neighborhood had guns at home, would that make your neighborhood a safer place to be, or a more dangerous place to be?” For each question, response options were: “safer,” “more dangerous,” “it depends,” or “don’t know.” We cross-classified gun owners into a priori risk perception groups based on their pattern of responses to questions (1) and (2) above, in the context of theory and past research indicating meaningful heterogeneity in gun owners’ perceptions of risk (Pallin et al., 2021; Salhi et al., 2021). Definitions and operationalizations of each group can be found in the Supplemental Appendix and are described here. The *Optimism Bias* group comprises gun owners who believed household gun ownership is comparatively safer for themselves than for similar others, which included those who said (1) having a gun at home made their home “safer” and (2) having guns at all homes in their neighborhood would make their neighborhood “more dangerous,” “it depends,” or “don’t know,” or who said (1) “it depends” or “don’t know” whether a gun at home made their home safer and (2) having guns at all homes in their neighborhood would make their neighborhood “more dangerous.” The *Always Safer* group comprises gun owners who unequivocally believed guns increase safety, that is, those who said (1) having a gun at home made their home “safer” and (2) having guns at all homes in their neighborhood would make their neighborhood “safer.” The *Uncertain* group included those who said (1) “it depends” or “don’t know” whether a gun at home made their home safer and (2) “it depends” or “don’t know” whether guns at all homes in their neighborhood would make their neighborhood safer. The *Other* group comprises gun owners with two distinct risk perceptions patterns, which were combined due to their infrequency. The first pattern reflects gun owners who unequivocally thought guns increase dangerousness (the opposite of the *Always Safer* group), that is, those who said (1) having a gun at home made their home “more dangerous” and (2) having guns at all homes in the neighborhood would make their neighborhood “more dangerous.” The second pattern reflects gun owners who believed household gun ownership is comparatively more dangerous for themselves than for similar others (the opposite of the *Optimism Bias* group), which included those who said (1) having a gun at home made their home “more dangerous” and (2) having guns at all homes in their neighborhood would make their neighborhood “safer,” “it depends,” or “don’t know,” or who said (1) “it depends” or “don’t know” whether a gun at home made their home safer and (2) having guns at all homes in their neighborhood would make their neighborhood “safer.” Respondents with missing data on risk perceptions were excluded from analysis (*n* = 5).

#### Sociodemographic Characteristics

A series of sociodemographic covariates, including gender, age, race and ethnicity, educational attainment, household income, marital status, and the presence of children under 18 years of age in the home, were collected as part of ongoing KP panel membership and merged with individual survey responses. Due to small cell sizes in some subgroups of gun owners, we collapsed ages 18 to 29 years and 30 to 44 years into a single category, and respondents who identified as Black, Hispanic/Latinx, multiracial, or of some “other” race were grouped together in a “minoritized racial or ethnic group” category. Urbanicity/rurality of residential census tracts was determined based on USDA Economic Research Service Rural-Urban Commuting Area (RUCA) codes and collapsed due to limited sample size in rural areas (California is a predominantly urban state) into two categories: “urban core” and “suburban or rural.” The prevalences of these sociodemographic variables in their original (disaggregated) form have been previously published (Kravitz-Wirtz et al., 2020).

#### Gun Ownership-Related Characteristics and Behaviors

We determined firearm ownership using two questions: (1) “Do you or does anyone you live with currently own any type of gun?” and (2) “Do you personally own a gun?” Respondents who answered both questions affirmatively were classified as gun owners and then asked a series of follow-up questions to identify the types of firearms they owned, reasons for ownership, firearm storage practices, length of firearm ownership, and frequency of firearm carrying in the past 30 days. Responses to these follow-up questions were dichotomized, consistent with previous research (Mauri et al., 2019; Salhi et al., 2021; Schleimer et al., 2021; Wertz et al., 2018).

#### Support for Selected Firearm Injury Prevention Policy Proposals and Interventions

Respondents were asked whether they would “support” or “oppose” three firearm policy proposals that were identified because they were under consideration by the state legislature or the court system at the time of the survey: (1) a 5-year prohibition on firearm purchasing and possession for people who had two driving under the influence (DUI) convictions in 5 years; (2) an amnesty program that would allow people to turn in high-capacity magazines (>10 rounds) no questions asked; and (3) a buyback program that would allow people to turn in high-capacity magazines in exchange for a small payment or reward. We also measured respondents’ level of support for two firearm injury prevention interventions by asking how often (“never,” “sometimes,” “usually,” or “always”) they thought it was appropriate: (1) “for doctors or other health professionals to talk to their patients about gun safety” and (2) “for parents to ask if there are unlocked guns in a home where their children go to play.” Consistent with past research, we collapsed “sometimes,” “usually,” and “always” into one category (Pallin et al., 2019). For each proposed policy or intervention, respondents could also select “don’t know.”

### Statistical Analysis

Reponses were weighted such that estimates are representative of the adult population of gun owners in California. The final weight variable was provided by Ipsos and combined pre-sample and study-specific post-stratification weights to account for sample design and survey non-coverage and non-response. Weighted percentages and their 95% confidence intervals (CIs) for each measure or cross-tabulation of measures were calculated using the *svy* and weighting commands in Stata, v15.1.

We used weighted multinomial logistic regression to examine whether gun owners classified with *Optimism Bias* differed from those in the *Always Safer*, *Uncertain*, and *Other* groups, separately, in their sociodemographic characteristics, gun ownership-related characteristics and behaviors, and support for each firearm injury prevention policy or intervention. Sociodemographic characteristics were included as covariates in models assessing gun-related characteristics and behaviors and support for policies and interventions. The *margins* and *nlcom* commands were used to generate *p* values. To adjust for multiple testing, we generated sharpened *q* values using Benjamini, Krieger, and Yekutieli’s (2006) two-stage procedure for controlling the false discovery rate (FDR; Benjamini et al., 2006). All statistical tests were two-tailed and *q* < .05 indicated statistical significance.

## Results

### Sociodemographic Characteristics

A plurality of gun owners belonged to the *Always Safer* group (40.9%; 95% CI = [34.0, 48.1]), 26.2% (95% CI = [20.6, 32.6]) of gun owners were classified as having *Optimism Bias*, 22.3% (95% CI = [16.5, 29.4]) of gun owners were classified in the *Uncertain* group, and the remaining 10.6% (95% CI = [6.8, 16.3]) of gun owners comprised the *Other* group (Table 1). The more than 1 in 4 gun owners classified with *Optimism Bias* were more often female (40.5%; 95% CI = [28.9, 53.3]), from minoritized racial or ethnic groups (44.7%; 95% CI = [31.9, 58.3]), and residents of suburban or rural areas (21.1%; 95% CI = [13.2, 31.9]) compared with those in the other risk perception groups (Table 2). They were also the least likely to have household incomes of $100,000 or more (43.4%; 95% CI = [30.8, 56.8]). Compared with the *Uncertain* group, gun owners classified with *Optimism Bias* were younger, more often married (75.9%; 95% CI = [65.3, 84.1]), and less often had a Bachelor’s degree (19.9%; 95% CI = [12.5, 30.2]) or advanced degree (11.4%; 95% CI = [6.5, 19.3]). The *Optimism Bias* group had children in their household (17.0%; 95% CI = [9.4, 28.7]) less often than the *Always Safer* and *Uncertain* groups, but more often than the *Other* group. These differences were not statistically significant after controlling for multiple testing.

**Table 1.** Crosstabulation of Gun Owners’ Risk Perceptions About Household Gun Ownership for Themselves (Rows) and for Others (Columns) (n = 424).

	If everyone in your neighborhood had guns at home, would that make your neighborhood . . .?				
Does having a gun at your home make it . . .?	Safer *N* (%) [95% CI]	More dangerous *N* (%) [95% CI]	It depends *N* (%) [95% CI]	Don’t know *N* (%) [95% CI]	Total
Safer	166 (40.9%) [34.0, 48.1]^a^	10 (3.2%) [0.9, 10.4]^b^	60 (12.2%) [8.8, 16.7]^b^	24 (4.4%) [2.7, 7.1]^b^	260 (60.7%) [53.3, 67.6]
More dangerous	2 (0.5%) [0.1, 2.6]^d^	17 (3.9%) [2.0, 7.6]^d^	0^d^	0^d^	19 (4.5%) [2.4, 8.2]
It depends	13 (4.0%) [1.7, 8.9]^d^	19 (3.4%) [1.8, 6.1]^b^	30 (8.6%) [4.7, 15.0]^c^	16 (5.8%) [2.7, 11.8]^c^	78 (21.7%) [15.7, 29.2]
Don’t know	10 (2.2%) [0.8, 6.1]^d^	14 (3.0%) [1.5, 6.0]^b^	6 (0.7%) [0.3, 1.7]^c^	37 (7.3%) [4.8, 10.8]^c^	67 (13.2%) [9.5, 18.0]
Total	191 (47.6%) [40.5, 54.8]	60 (13.5%) [9.1, 19.6]	96 (21.5%) [16.1, 28.0]	77 (17.4%) [12.7, 23.3]	424 (100.0%)

*Note.* All percentages are weighted. Respondents were cross-classified into four risk perceptions groups based on their pattern of responses to both questions.

a Belongs to *Always Safer* group. ^b^Belongs to *Optimism Bias* group. ^c^Belongs to *Uncertain* group. ^d^Belongs to *Other* group.

**Table 2.** Sociodemographic Characteristics of Gun Owners, Overall and by Risk Perception Group (n = 424).

Characteristic	All gun owners	Optimism bias	Always safer	Uncertain	Other		FDR *q* value
	*N* (%) [95% CI]	*N* (%) [95% CI]	*N* (%) [95% CI]	*N* (%) [95% CI]	*N* (%) [95% CI]	*p* value	
Gender							
Male	298 (73.4%) [67.3, 78.7]	81 (59.5%)[46.7, 71.1]	119 (81.5%)[73.9, 87.2]	63 (74.7%)[60.9, 84.9]	35 (73.8%)[47.8, 89.6]	.002^a^ .085^b^ .263^c^	.129^a^ .680^b^ 1.000^c^
Female	126 (26.6%) [21.3, 32.7]	46 (40.5%)[28.9, 53.3]	47 (18.5%) [12.8, 26.1]	26 (25.3%)[15.1, 39.1]	7 (26.2%)[10.4, 52.2]		
Age							
18–44	52 (24.2%) [18.0, 31.8]	16 (22.5%) [13.3, 35.4]	24 (27.8%) [17.8, 40.7]	6 (13.2%) [4.9, 30.9]	6 (37.6%) [17.7, 62.7]	.512^a^ .271^b^ .265^c^	1.000^a^ 1.000^b^ 1.000^c^
45–59	98 (33.3%) [26.5, 40.9]	30 (30.2%)[18.6, 45.0]	41 (34.3%) [24.3, 46.0]	18 (35.5%)[20.8, 53.6]	9 (32.5%)[14.4, 58.0]	.643^a^ .635^b^ .867^c^	1.000^a^ 1.000^b^ 1.000^c^
60+	274 (42.5%) [35.9, 49.4]	81 (47.3%)[35.1, 59.8]	101 (37.8%)[28.8, 47.8]	65 (51.4%)[34.8, 67.6]	27 (29.9%)[16.6, 47.7]	.242^a^ .706^b^ .094^c^	1.000^a^ 1.000^b^ .680^c^
Race							
White	315 (64.3%) [56.5, 71.5]	89 (55.3%)[41.7, 68.1]	127 (72.3%)[60.5, 81.7]	65 (56.7%)[38.9, 72.9]	34 (71.6%)[46.4, 88.1]	.053^a^ .903^b^ .212^c^	.661^a^ 1.000^b^ 1.000^c^
Minoritized racial or ethnic group	109 (35.7%) [28.6, 43.5]	38 (44.7%) [31.9, 58.3]	39 (27.7%) [18.3, 39.4]	24 (43.3%) [27.1, 61.1]	8 (28.4%) [11.9, 53.6]		
Education							
High school or less	53 (25.4%) [18.8, 33.3]	16 (26.2%) [14.5, 42.5]	21 (24.3%) [15.1, 36.6]	10 (22.3%) [10.7, 40.8]	6 (33.9%) [14.6, 60.5]	.834^a^ .715^b^ .597^c^	1.000^a^ 1.000^b^ 1.000^c^
Some college	171 (42.4%) [35.5, 49.6]	52 (42.4%) [30.8, 55.0]	68 (46.5%) [35.7, 57.7]	37 (39.9%) [25.0, 57.0]	14 (31.6%) [15.6, 53.5]	.630^a^ .810^b^ .359^c^	1.000^a^ 1.000^b^ 1.000^c^
Bachelor’s degree	113 (18.8%) [14.2, 24.4]	38 (19.9%) [12.5, 30.2]	44 (15.7%) [10.4, 23.1]	19 (24.4%) [12.3, 42.6]	12 (15.7%) [7.2, 31.1]	.447^a^ .623^b^ .575^c^	1.000^a^ 1.000^b^ 1.000^c^
Advanced degree	87 (13.5%) [10.0, 17.9]	21 (11.4%) [6.5, 19.3]	33 (13.4%) [8.5, 20.5]	23 (13.4%) [7.4, 23.0]	10 (18.9%) [6.4, 44.2]	.647^a^ .699^b^ .462^c^	1.000^a^ 1.000^b^ 1.000^c^
Income							
Less than $25,000	31 (5.3%) [3.3, 8.6]	14 (7.4%) [3.5, 15.0]	9 (5.3%) [2.1, 12.5]	7 (5.2%) [2.1, 12.2]	1 (0.8%) [0.1, 5.5]	.554^a^ .528^b^ .020^c^	1.000^a^ 1.000^b^ .661^c^
$25,000–$59,999	108 (21.3%) [16.3, 27.3]	29 (18.8%) [11.7, 28.9]	40 (23.4%) [14.9, 34.6]	27 (16.6%) [9.8, 26.7]	12 (29.2%) [13.6, 51.8]	.499^a^ .715^b^ .348^c^	1.000^a^ 1.000^b^ 1.000^c^
$60,000–$99,999	127 (20.0%) [15.8, 25.0]	40 (30.4%) [20.3, 42.7]	49 (16.8%) [11.6, 23.7]	27 (16.0%) [9.0, 26.6]	11 (15.2%) [7.1, 29.4]	.038^a^ .048^b^ .058^c^	.661^a^ .661^b^ .661^c^
$100,000 or more	158 (53.4%) [46.3, 60.3]	44 (43.4%) [30.8, 56.8]	68 (54.6%) [43.6, 65.1]	28 (62.3%) [47.5, 75.0]	18 (54.9%) [33.1, 74.9]	.201^a^ .055^b^ .384^c^	1.000^a^ .661^b^ 1.000^c^
Marital status							
Married or living with partner	286 (70.9%) [63.8, 77.1]	84 (75.9%) [65.3, 84.1]	117 (72.0%) [60.3, 81.4]	52 (58.1%) [40.8, 73.5]	33 (81.2%) [60.7, 92.4]	.591^a^ .071^b^ .571^c^	1.000^a^ .661^b^ 1.000^c^
Widowed	29 (3.9%) [2.5, 6.0]	11 (6.3%) [3.0, 12.7]	6 (2.3%) [0.9, 5.9]	8 (4.2%) [1.8, 9.5]	4 (2.9%) [0.9, 8.6]	.121^a^ .479^b^ .223^c^	.778^a^ 1.000^b^ 1.000^c^
Divorced or separated	71 (13.2%) [9.0, 18.8]	19 (6.7%) [3.9, 11.5]	32 (14.8%) [8.6, 24.2]	17 (20.5%) [9.6, 38.5]	3 (7.5%) [2.1, 23.5]	.064^a^ .069^b^ .884^c^	.661^a^ .661^b^ 1.000^c^
Never married	38 (12.1%) [7.6, 18.7]	13 (11.0%) [5.4, 21.3]	11 (10.9%) [4.6, 23.6]	12 (17.2%) [7.1, 35.9]	2 (8.5%) [1.7, 32.5]	.979^a^ .454^b^ .734^c^	1.000^a^ 1.000^b^ 1.000^c^
Children under 18 in household							
Yes	48 (18.3%) [13.1, 24.9]	13 (17.0%)[9.4, 28.7]	25 (21.4%) [13.4, 32.3]	8 (18.1%) [7.5, 37.7]	2 (10.0%) [2.2, 35.6]	.524^a^ .898^b^ .426^c^	1.000^a^ 1.000^b^ 1.000^c^
No	376 (81.7%) [75.1, 86.9]	114 (83.0%)[71.4, 90.6]	141 (78.7%)[67.7, 86.6]	81 (81.9%)[62.3, 92.5]	40 (90.0%) [64.4, 97.8]		
Urbanicity							
Urban core	344 (83.6%) [78.3, 87.7]	99 (78.9%) [68.1, 86.8]	136 (85.0%)[77.1, 90.5]	75 (86.9%)[71.6, 94.5]	34 (82.4%)[62.6, 92.9]	.302^a^ .280^b^ .698^c^	1.000^a^ 1.000^b^ 1.000^c^
Suburban or rural	79 (16.5%) [12.3, 21.7]	27 (21.1%) [13.2, 31.9]	30 (15.0%) [9.5, 22.9]	14 (13.1%) [5.5, 28.4]	8 (17.6%) [7.1, 37.4]		

*Note.* All percentages are weighted. The *Optimism Bias* group includes respondents who said having a gun at home made their home “safer” and having guns at all homes in their neighborhood would make their neighborhood “more dangerous,” “it depends,” or “don’t know,” or who said “it depends” or “don’t know” whether a gun at home made their home safer and having guns at all homes in their neighborhood would make their neighborhood “more dangerous.” The *Always Safer* group includes respondents who said having a gun at home made their home “safer” and having guns at all homes in their neighborhood would make their neighborhood “safer.” The *Uncertain* group includes respondents who said “it depends” or “don’t know” whether a gun at home made their home safer and “it depends” or “don’t know” whether guns at all homes in their neighborhood would make their neighborhood safer. The *Other* group includes respondents who said having a gun at home made their home “more dangerous” and having guns at all homes in their neighborhood would make their neighborhood “safer,” “more dangerous,” “it depends,” or “don’t know,” or who said “it depends” or “don’t know” whether a gun at home made their home safer and having guns at all homes in their neighborhood would make their neighborhood “safer.”

a *Optimism Bias* group versus *Always Safer* group. ^b^*Optimism Bias* group versus *Uncertain* group. ^c^*Optimism Bias* group versus *Other* group.

### Gun Ownership-Related Characteristics and Behaviors

Approximately 13.0% (95% CI = [6.5, 25.0]) of gun owners classified with *Optimism Bias* were new owners who had lived in a household without firearms at some point in the past 5 years and had since acquired their firearm(s) (Table 3). This was the largest percentage of any group. Handgun ownership (85.8%; 95% CI = [76.9, 91.7]) and owning a gun for protection (62.0%; 95% CI = [49.6, 73.0]) were more common among gun owners in the *Optimism Bias* group than among those in the *Uncertain* and *Other* groups, but these percentages were similar to those in the *Always Safer* group. Gun owners in the *Optimism Bias* group were less likely than the *Always Safer* group, but more likely than the *Uncertain* group, to store at least one gun loaded and unlocked in or around their home (19.5%; 95% CI = [9.4, 36.0]) and to have carried a loaded handgun in the past 30 days (16.3%; 95% CI = [6.7, 34.7]). However, only the difference in reasons for ownership between the *Optimism Bias* and *Uncertain* groups was statistically significant after controlling for sociodemographic characteristics and multiple testing (*q* = .001).

**Table 3.** Gun-Related Characteristics and Behaviors Among Gun Owners, Overall and by Risk Perception Group (n = 424).

Characteristic	All gun owners	Optimism bias	Always safer	Uncertain	Other		
	*N* (%) [95% CI]	*N* (%) [95% CI]	*N* (%) [95% CI]	*N* (%) [95% CI]	*N* (%) [95% CI]	*p* value	FDR *q* value
Type of guns owned							
1+ handgun	342 (81.6%) [75.1, 86.6]	102 (85.8%)[76.9, 91.7]	146 (85.9%)[73.6, 93.0]	65 (74.9%)[58.7, 86.2]	29 (68.3%) [44.9, 85.0]	.595^a^ .139^b^ .166^c^	.838^a^ .556^b^ .556^c^
Long guns only	82 (18.5%) [13.4, 24.9]	25 (14.2%) [8.3, 23.2]	20 (14.1%) [7.0, 26.4]	24 (25.1%)[13.8, 41.3]	13 (31.7%) [15.0, 55.1]		
Reasons for ownership							
Owns 1+ gun primarily for protection	216 (49.5%) [42.2, 56.7]	74 (62.0%)[49.6, 73.0]	103 (61.2%)[49.6, 71.6]	24 (21.0%)[11.9, 34.3]	15 (34.3%)[16.1, 58.6]	.785^a^ <.001^b^ .026^c^	1.000^a^ .**001**^b^ .352^c^
Owns for other reasons only	202 (50.5%) [43.3, 57.8]	52 (38.0%)[27.0, 50.4]	58 (38.8%) [28.4, 50.4]	65 (79.0%)[65.7, 88.1]	27 (65.7%)[41.4, 83.9]		
Storage practices							
No guns loaded and unlocked in/around home	306 (73.6%) [66.3, 79.8]	92 (73.6%) [60.4, 86.8]	108 (67.5%) [56.5, 78.4]	72 (86.8%) [78.4, 95.1]	34 (69.2%) [43.7, 94.7]	.439^a^ .107^b^ .966^c^	.783^a^ .556^b^ 1.000^c^
1+ gun loaded and unlocked in/around home	78 (18.4%) [13.0, 25.4]	24 (19.5%) [9.4, 36.0]	39 (24.0%) [15.5, 35.4]	10 (7.0%)[2.9, 15.6]	5 (18.0%) [4.4, 50.8]	.631^a^ .068^b^ .615^c^	.838^a^ .478^b^ .838^c^
Any guns stored at work/somewhere else^d^	31 (8.0%) [4.9, 12.8]	10 (7.0%) [3.3, 13.9]	13 (8.5%) [3.9, 17.6]	6 (6.3%) [2.4, 15.3]	2 (12.8%) [2.6, 44.9]	.521^a^ .978^b^ .486^c^	.824^a^ 1.000^b^ .823^c^
Length of ownership							
New owner (≤5 years)	28 (8.9%) [5.6, 13.7]	11 (13.2%) [6.5, 25.0]	7 (6.4%) [2.7, 14.6]	8 (8.8%)[3.5, 20.1]	2 (7.4%) [1.3, 32.8]	.122^a^ .412^b^ .282^c^	.556^a^ .783^b^ .773^c^
Longstanding owner (>5 years)	394 (91.1%) [86.4, 94.4]	116 (86.8%) [75.0, 93.5]	157 (93.6%) [85.5, 97.3]	81 (91.2%) [79.9, 96.5]	40 (92.6%) [67.2, 98.7]		
Carried loaded handgun in past 30 days							
Yes	47 (17.2%) [11.8, 24.4]	11 (16.3%) [6.7, 34.7]	31 (27.0%) [17.6, 39.1]	2 (3.2%) [0.7, 13.4]	3 (11.1%) [2.8, 35.3]	.337^a^ .062^b^ .362^c^	.773^a^ .478^b^ .773^c^
No	374 (82.8%) [75.6, 88.2]	116 (83.7%) [65.3, 93.3]	132 (73.0%) [60.9, 82.4]	87 (96.8%) [86.6, 99.3]	39 (88.9%) [64.7, 97.2]		

*Note.* All percentages are weighted. Models adjusted for gender, age, race, education, income, marital status, presence of children in the household, and urbanicity. Boldface indicates statistical significance (*q* < .05). The *Optimism Bias* group includes respondents who said having a gun at home made their home “safer” and having guns at all homes in their neighborhood would make their neighborhood “more dangerous,” “it depends,” or “don’t know,” or who said “it depends” or “don’t know” whether a gun at home made their home safer and having guns at all homes in their neighborhood would make their neighborhood “more dangerous.” The *Always Safer* group includes respondents who said having a gun at home made their home “safer” and having guns at all homes in their neighborhood would make their neighborhood “safer.” The *Uncertain* group includes respondents who said “it depends” or “don’t know” whether a gun at home made their home safer and “it depends” or “don’t know” whether guns at all homes in their neighborhood would make their neighborhood safer. The *Other* group includes respondents who said having a gun at home made their home “more dangerous” and having guns at all homes in their neighborhood would make their neighborhood “safer,” “more dangerous,” “it depends,” or “don’t know,” or who said “it depends” or “don’t know” whether a gun at home made their home safer and having guns at all homes in their neighborhood would make their neighborhood “safer.”

a *Optimism Bias* group versus *Always Safer* group. ^b^*Optimism Bias* group versus *Uncertain* group. ^c^*Optimism Bias* group versus *Other* group. ^d^Respondents who stored any firearm at work or someplace else had to be categorized separately due to an error in the survey design that precluded us from accounting for all the ways in which firearms were stored in and around the home for these respondents.

### Support for Selected Firearm Injury Prevention Policies and Interventions

Gun owners classified with *Optimism Bias* demonstrated higher levels of support for 4 of the 5 proposed firearm policies and interventions than those in the *Always Safer* and *Uncertain* groups (Table 4). More than 3 in 5 gun owners in the *Optimism Bias* group supported a firearm prohibition for people with two or more DUI convictions (63.5%; 95% CI = [50.8, 74.5]), an amnesty program for high-capacity magazines (61.8%; 95% CI = [49.4, 72.9]), and a buyback program for high-capacity magazines (67.8%; 95% CI = [55.5, 78.1]); more than half (55.9%; 95% CI = [42.5, 68.5]) thought it was at least sometimes appropriate for doctors to talk with their patients about gun safety. Nearly 8 in 10 (79.7%; 95% CI = [68.9, 87.4]) gun owners classified with *Optimism Bias*—the lowest of any group—thought it was at least sometimes appropriate for parents to ask about the presence of unlocked guns in homes where their children play; they were the most likely of any group to respond “don’t know” (13.7%; 95% CI = [7.4, 24.1]). After controlling for sociodemographic characteristics and multiple testing, only the differences in support (*q* = .048) and opposition (*q* = .048) for the amnesty program between the *Optimism Bias* and *Always Safer* groups were statistically significant.

**Table 4.** Support for Policies and Interventions Among Gun Owners, Overall and by Risk Perception Group (n = 424).

Intervention	All gun owners	Optimism bias	Always safer	Uncertain	Other		
	*N* (%) [95% CI]	*N* (%) [95% CI]	*N* (%) [95% CI]	*N* (%) [95% CI]	*N* (%) [95% CI]	*p* value	FDR *q* value
Firearm prohibition for 2+ DUI convictions							
Support	247 (49.9%) [42.7, 57.1]	87 (63.5%) [50.8, 74.5]	85 (46.3%) [35.6, 57.3]	49 (44.7%) [29.5, 61.0]	26 (40.8%) [22.4, 62.1]	.070^a^ .141^b^ .074^c^	.370^a^ .452^b^ .370^c^
Oppose	109 (31.5%) [25.0, 38.8]	26 (22.7%) [14.2, 34.1]	53 (34.6%) [25.0, 45.7]	18 (27.2%) [14.2, 45.9]	12 (50.2%) [28.6, 71.8]	.254^a^ .916^b^ .047^c^	.647^a^ 1.000^b^ .355^c^
Don’t know	68 (18.6%) [13.2, 25.7]	14 (13.8%) [7.0, 25.4]	28 (19.1%) [10.8, 31.5]	22 (28.0%) [14.8, 46.7]	4 (9.0%) [3.1, 23.1]	.294^a^ .098^b^ .617^c^	.708^a^ .417^b^ 1.000^c^
Amnesty program for high-capacity magazines							
Support	232 (51.0%) [43.8, 58.2]	81 (61.8%) [49.4, 72.9]	64 (36.7%) [27.0, 47.7]	58 (56.8%) [39.5, 72.5]	29 (67.1%) [42.1, 85.1]	.002^a^ .622^b^ .726^c^	.**048**^a^ 1.000^b^ 1.000^c^
Oppose	110 (29.1%) [22.9, 36.3]	20 (18.0%) [10.9, 28.3]	69 (42.7%) [32.3, 53.8]	14 (20.1%) [8.5, 40.6]	7 (22.9%) [7.5, 51.9]	.001^a^ .942^b^ .661^c^	.**048**^a^ 1.000^b^ 1.000^c^
Don’t know	80 (19.9%) [14.7, 26.3]	24 (20.2%) [12.2, 31.5]	33 (20.5%) [12.2, 32.4]	17 (23.1%) [12.1, 39.3]	6 (10.1%) [3.9, 23.5]	.779^a^ .559^b^ .158^c^	1.000^a^ .966^b^ .484^c^
Buyback program for high-capacity magazines							
Support	247 (55.2%) [47.9, 62.3]	90 (67.8%) [55.5, 78.1]	77 (46.8%) [36.1, 57.9]	56 (52.6%) [35.9, 68.7]	24 (61.8%) [38.2, 80.9]	.012^a^ .266^b^ .512^c^	.113^a^ .647^b^ .966^c^
Oppose	96 (22.8%) [17.4, 29.1]	18 (14.6%) [8.3, 24.4]	59 (34.2%) [24.7, 45.2]	11 (11.5%) [5.4, 22.7]	8 (22.4%) [7.3, 51.6]	.010^a^ .415^b^ .475^c^	.113^a^ .837^b^ .905^c^
Don’t know	78 (22.1%) [16.2, 29.3]	18 (17.6%) [9.9, 29.2]	29 (19.0%) [10.9, 31.0]	21 (36.0%) [20.8, 54.5]	10 (15.8%) [7.3, 31.1]	.708^a^ .081^b^ .971^c^	1.000^a^ .370^b^ 1.000^c^
Doctors talking about gun safety							
At least sometimes appropriate	225 (52.8%) [45.5, 59.9]	78 (55.9%) [42.5, 68.5]	72 (48.2%) [37.4, 59.2]	46 (49.3%) [33.1, 65.7]	29 (69.6%) [45.5, 86.2]	.231^a^ .472^b^ .534^c^	.647^a^ .905^b^ .966^c^
Never appropriate	164 (40.0%) [33.0, 47.5]	38 (34.3%) [22.4, 48.6]	88 (50.3%) [39.3, 61.2]	28 (36.2%) [21.5, 54.0]	10 (22.7%) [8.9, 47.0]	.058^a^ .834^b^ .370^c^	.370^a^ 1.000^b^ .800^c^
Don’t know	35 (7.2%) [4.5, 11.4]	11 (9.8%) [4.0, 21.8]	6 (1.5%) [0.5, 4.5]	15 (14.4%) [7.4, 26.2]	3 (7.7%) [1.4, 32.3]	.034^a^ .315^b^ .688^c^	.294^a^ .734^b^ 1.000^c^
Parents asking about unlocked guns							
At least sometimes appropriate	347 (85.3%) [80.5, 89.0]	101 (79.7%) [68.9, 87.4]	135 (86.3%) [78.7, 91.5]	76 (88.6%) [77.7, 94.6]	35 (88.1%) [68.0, 96.3]	.383^a^ .189^b^ .424^c^	.800^a^ .553^b^ .837^c^
Never appropriate	37 (7.9%) [5.3, 11.8]	9 (6.6%) [3.1, 13.4]	22 (11.3%) [6.6, 18.7]	4 (3.7%) [1.1, 11.8]	2 (7.3%) [1.2, 32.9]	.112^a^ .558^b^ .621^c^	.437^a^ .966^b^ 1.000^c^
Don’t know	36 (6.8%) [4.4, 10.3]	16 (13.7%) [7.4, 24.1]	7 (2.4%) [1.0, 5.8]	8 (7.7%) [3.1, 17.8]	5 (4.6%) [1.7, 12.1]	.005^a^ .255^b^ .083^c^	.078^a^ .647^b^ .370^c^

*Note.* All percentages are weighted. Models adjusted for gender, age, race, education, income, marital status, presence of children in the household, and urbanicity. Boldface indicates statistical significance (*q* < .05). The *Optimism Bias* group includes respondents who said having a gun at home made their home “safer” and having guns at all homes in their neighborhood would make their neighborhood “more dangerous,” “it depends,” or “don’t know,” or who said “it depends” or “don’t know” whether a gun at home made their home safer and having guns at all homes in their neighborhood would make their neighborhood “more dangerous.” The *Always Safer* group includes respondents who said having a gun at home made their home “safer” and having guns at all homes in their neighborhood would make their neighborhood “safer.” The *Uncertain* group includes respondents who said “it depends” or “don’t know” whether a gun at home made their home safer and “it depends” or “don’t know” whether guns at all homes in their neighborhood would make their neighborhood safer. The *Other* group includes respondents who said having a gun at home made their home “more dangerous” and having guns at all homes in their neighborhood would make their neighborhood “safer,” “more dangerous,” “it depends,” or “don’t know,” or who said “it depends” or “don’t know” whether a gun at home made their home safer and having guns at all homes in their neighborhood would make their neighborhood “safer.”

a *Optimism Bias* group versus *Always Safer* group. ^b^*Optimism Bias* group versus *Uncertain* group. ^c^*Optimism Bias* group versus *Other* group.

## Discussion

This state-representative general population survey of adult Californians examined comparative risk perceptions of household firearm ownership among firearm owners, more specifically comparing beliefs about the risks and benefits associated with their own behavior (e.g., having a gun at their own home) to those about the risks and benefits associated with other people engaging in the same behavior (e.g., having guns at all homes in their neighborhood). Past research on other health risk scenarios, including driving and motor vehicle collisions or masking and COVID-19 infection, suggests that people tend to interpret the risks and benefits associated with undertaking various health-related actions in unrealistically optimistic or self-serving ways by underrating their own vulnerability to harmful events while perceiving others to be more susceptible and vice versa for positive events; a concept referred to as optimism bias (White et al., 2004; Wise et al., 2020). Past research by the authors has demonstrated that optimism bias is likewise present in public perceptions of household firearm ownership, regardless of respondents’ gun ownership status (Aubel et al., 2023), while another study has also found that, across racial and ethnic identities, gun owners are more optimistic about the safety benefits of their own gun ownership than gun ownership and gun carrying among others (Ward et al., 2023). However, this is the first study, to our knowledge, to empirically examine the potential implications of optimism bias for firearm injury risk and prevention.

Similar to past research, we hypothesized that optimism bias in firearm-related risk perceptions would influence the adoption of risk reduction and preventive practices. For example, recent research has found that individuals with COVID-19 pandemic-related optimism bias, who perceive their personal risk of COVID-19 infection as being lower than the average person, are less likely to engage in health protective behaviors such as increased hand washing, mask wearing, and social distancing (Fragkaki et al., 2021). In the current study examining household firearm ownership, few observed differences in sociodemographic or gun ownership and use characteristics, nor support for selected firearm injury prevention interventions, between firearm owners with optimism bias and those with other patterns of risk perceptions achieved statistical significance; however, and markedly, sizable differences among risk perception groups in the estimated frequency of several such characteristics and opinions deserve further comment.

Compared with the plurality (41%) of gun owners who unequivocally said guns increase safety when asked both about guns at their own home and guns at all homes in their neighborhood, for example, the roughly 1 in 4 gun owners with optimism bias were more often female (41% vs. 19%) and more often members of minoritized racial or ethnic groups (45% vs. 28%). They also were more often new owners (13% vs. 6%), having acquired their firearm(s) within the past 5 years, less often carried a firearm in the past 30 days (16% vs. 27%) and, despite similar levels of reported ownership for protection, less often stored firearms using the least secure method, loaded and not locked up (20% vs. 24%). In addition, and similarly, gun owners with optimism bias more often endorsed support for 4 of 5 firearm injury prevention policy proposals and interventions than those in the *Always Safer* group, including DUI-related prohibitions on firearm ownership (64% vs. 46%), large-capacity magazine relinquishment (62% vs. 37%) and buyback (68% vs. 47%) programs, and gun safety conversations between doctors and patients (56% vs. 48%). Support for parents asking about the presence of unlocked guns in homes where their children play was high among all groups (≥80%), though, relatively less so among gun owners with optimism bias.

Thus, relative to the plurality of gun owners who viewed household gun ownership as always safer, owners with optimism bias more often resembled more recent demographic and ideological shifts observed in firearm ownership, especially during the firearm purchasing surge starting amid the COVID-19 pandemic in 2020, wherein increasing percentages of (often new) firearm owners identified as female or people of color (Lyons et al., 2020; Miller et al., 2022) and for whom past research has further documented higher levels of support for a broader array of preventive policies that traditionally have been less popular among conventional (older, White, male, politically conservative) firearm owners (e.g., assault weapons and large-capacity magazine bans and minimum purchasing age requirements; Crifasi et al., 2021). Accordingly, whereas interpreting risks and benefits in unrealistically optimistic or self-serving ways has been associated in past research with less adherence and support for preventive action in many other (though not all) health domains (Ferrer & Klein, 2015), in this novel case of household firearm ownership, acknowledging some level of risk associated with having guns at home, even if only or more so for others in one’s neighborhood than for oneself, may instead be indicative of greater receptivity to firearm injury prevention and risk reduction efforts, or a “movable middle” subgroup of gun owners.

From this perspective, gun owners with optimism bias, and more generally, those whose risk perceptions are conditional or undetermined (vs. fixed or ideological in nature), may be critical partners in developing and delivering credible, relevant, and meaningful messaging on firearm safety and injury prevention strategies. For instance, past research has documented that gun owners who say “it depends” when asked whether guns make homes safer or more dangerous tend to condition their risk perceptions on whether gun owners are perceived as knowledgeable about and proficient in using guns safely, suggesting that these individuals may be especially receptive to, or even advocates for, injury prevention efforts that emphasize ongoing training and tools for safe firearm use and storage (Pallin et al., 2021). In the current study, gun owners who consistently said “it depends” or “don’t know” (the *Uncertain* group) demonstrated the lowest prevalence of personal risk behaviors, such as storing firearms loaded and unlocked (7%) and carrying a loaded firearm in public (3%). Notably, however, they were also more often undecided in their public policy opinions relative to the *Optimism Bias* group. Additional research to increase understanding of the underlying similarities and differences between these groups, including their predilection for individual versus population-level prevention efforts, could provide valuable insights for engaging gun owners in firearm injury prevention initiatives. Given the focus of this study, further investigation of support for a wider range of policies and interventions could also help ascertain whether gun owners with optimism bias are amenable to firearm injury prevention efforts in general or only the specific subset examined here.

More broadly, as an initial point of entry for processing risk information, firearm injury prevention efforts and coordinated risk communications should consider leveraging individuals’ tendencies to more readily recognize risk associated with the behavior of others, perhaps by inducing or reinforcing other-oriented firearm-related risk perceptions and a collective sense of responsibility for firearm safety. Incoming information framed in relation to others (e.g., peers, neighbors), especially when the “other” belongs to the same social group as the message recipient (e.g., a fellow firearm owner), may be perceived as less threatening to the symbolic importance often assigned to guns in conceptualizing one’s own identity and sense of self, as well as broader attitudes not just about what guns do but rather, and significantly, what guns mean in individuals’ lives (Metzl, 2019; Warner & Ratcliff, 2021). To extend these findings further, future research considering not only the reference point of risk communication messages (self vs. other) but also the intersection of reference point with preferences for and effectiveness of different potential “credible” messengers, including across demographic lines such as age, sex, and race or ethnicity, is warranted (Anestis et al., 2021).

Findings should be interpreted in light of several limitations. First, these findings may not generalize beyond California, which has relatively stringent firearm policies compared with other states. Second, the cross-sectional nature of the study precludes us from making inferences about causation or the directionality of relationships (e.g., whether perceptions about the risks and benefits incurred through firearm ownership influence the decision to buy a gun for self-protection or vice versa). Third, despite collapsing categories, analyses may have lacked the statistical power needed to conclude differences between risk perception groups; collapsing categories may also have oversimplified gun owners’ characteristics, behaviors, and policy opinions. Fourth, as with most survey research, findings are subject to non-response and social desirability biases.

Finally, it is important to note that operationalizations of optimism bias vary, impacting interpretations and the ability to directly compare our findings with those of other studies. We defined optimism bias in comparative terms (i.e., underestimating risk for oneself vs. others). However, gun owners who unequivocally believe firearms increase safety despite empirical evidence suggesting otherwise could also be described as having unrealistic absolute optimism (i.e., underestimating risk for oneself vs. objective risk; Shepperd et al., 2015). Our operationalization of optimism bias, as well as the *Uncertain* and *Other* groups, which combined respondents with several different patterns of risk perceptions (e.g., those who said “safer” for themselves and “it depends” for others vs. those who said “don’t know” for themselves and “more dangerous” for others), may also have masked informative heterogeneity in gun owners’ perceptions of firearm-related risks.

## Conclusion

This study provides helpful new considerations for addressing perceptions and misperceptions about the risks and benefits of household firearm ownership and injury preventive actions. While the plurality of gun owners believed that having a gun at home unequivocally made it safer, those who acknowledged some level of risk or uncertainty associated with household firearms, even if only or more so for others in their neighborhood than for themselves, more often reported health protective behaviors and support for firearm injury prevention strategies. Accordingly, public health messaging and community-level interventions that elicit more general (or other-oriented) risk perceptions and promote a collective sense of responsibility rather than focusing solely or primarily on personal risk, especially interventions delivered by credible messengers, may enhance the effectiveness of strategies designed to reduce and prevent firearm-related harm.

## Supplemental Material

sj-docx-1-heb-10.1177_10901981241267212 – Supplemental material for Optimism Bias Among Gun Owners: Associations With Firearm Injury Prevention Practices and Policy Support Supplemental material, sj-docx-1-heb-10.1177_10901981241267212 for Optimism Bias Among Gun Owners: Associations With Firearm Injury Prevention Practices and Policy Support by Amanda J. Aubel, Garen J. Wintemute, Aaron B. Shev and Nicole Kravitz-Wirtz in Health Education & Behavior
