# Supplementary material for: Optimism Bias Among Gun Owners: Associations With Firearm Injury Prevention Practices and Policy Support
Source: Health Educ Behav. 2024 Jul 30;52(3):266–77. doi: 10.1177/10901981241267212 (PMC13009218; doi:10.1177/10901981241267212)
Supplement: sj-docx-1-heb-10.1177_10901981241267212 – Supplemental material for Optimism Bias Among Gun Owners: Associations With Firearm Injury Prevention Practices and Policy Support [file sj-docx-1-heb-10.1177_10901981241267212.docx]

Appendix for “Optimism Bias Among Gun Owners: Associations with Firearm Injury Prevention Practices and Policy Support”

Amanda J. Aubel, Garen J. Wintemute, Aaron B. Shev & Nicole Kravitz-Wirtz

Table. Definitions and operationalizations of risk perception groups among gun owners, 2018 California Safety and Wellbeing Survey

| **Name of group** | **Plain language definition** | **Operationalization** | |
| --- | --- | --- | --- |
|  |  | Response to “Does having a gun at your home make it…?” | Response to “If everyone in your neighborhood had guns at home, would that make your neighborhood…?” |
| *Always Safer* | Gun owners who unequivocally believe guns increase safety | Safer | Safer |
| *Optimism Bias* | Gun owners who believe household gun ownership is comparatively safer for themselves than for similar others | Safer | More dangerous |
|  |  | Safer | It depends |
|  |  | Safer | Don’t know |
|  |  | It depends | More dangerous |
|  |  | Don’t know | More dangerous |
| *Uncertain* | Gun owners whose risk perceptions about household firearm ownership are consistently undetermined or conditional on other factors | It depends | It depends |
|  |  | It depends | Don’t know |
|  |  | Don’t know | Don’t know |
|  |  | Don’t know | It depends |
| *Other* | Gun owners who unequivocally believe guns increase dangerousness | More dangerous | More dangerous |
|  | Gun owners who believe household gun ownership is comparatively more dangerous for themselves than for similar others | More dangerous | Safer |
|  |  | More dangerous | It depends |
|  |  | More dangerous | Don’t know |
|  |  | It depends | Safer |
|  |  | Don’t know | Safer |
